# Chapped Hands

**Published:** 1890-01

**Authors:** 


CHAPPED HANDS.—The following is a pleasant and efficacious application for chapped hands:

| | | |
|---|---|---|
| ℞ | Quince seed - - - | ½ ounce. |
| | Water - - - - - - - - - | q. s. |
| | Glycerine - - - - | 1 fluid ounce. |
| | Alcohol - - - - - - - - - | 4 fluid ounces. |

Macerate the quince seed with a pint of water for twenty-four hours, stirring frequently, strain with gentle pressure through muslin, and make up the volume to 1 pint with water; then add the glycerine and finally the alcohol containing the perfume, and stir briskly.—*Pharmaceutical Era*, Nov., 1889.

---

IT is reported in the St. Louis *Post-Dispatch* of October 15, that a woman living at Fort Smith, Arkansas, seventy-one years old, gave birth, on October 14, to a well-formed and healthy male child. Two years ago the woman, then a widow, married a young farm-hand employed by her. The case has caused a great deal of excitement among the neighboring physicians and it will be thoroughly investigated.

